# It Seems Italian, Doesn’t It? An Exploratory Analysis of English and Spanish Consumers about Italian Appearance Food Products

**DOI:** 10.3390/foods11101392

**Published:** 2022-05-11

**Authors:** Mariarosaria Simeone, Morena Cinquegrana, Carlo Russo

**Affiliations:** 1 Department of Law, Economics, Management and Quantitative Methods, University of Sannio, 82100 Benevento, Italy; 2 Business Management Made in Italy, Bologna Business School, University of Bologna, 40136 Bologna, Italy; morena.cinquegrana@studio.unibo.it; 3 Department of Economics and Law, University of Cassino and Lazio Meridionale, 03043 Cassino, Italy; carlo.russo@unicas.it

**Keywords:** country of origin, unfair practices, Italian sounding, food culture, food attributes

## Abstract

The Italian export of agri-food products has been increasingly threatened by the unfair use of misleading Italian symbols (such as the national flag or the green-white-red colors) by non-Italian producers. This research paper investigated what English and Spanish consumers know about “Made in Italy” food, and their attitude towards Italian appearance food products. Primary data were collected in Spain and England, and a probit model was used to identify the determinants of consumers’ vulnerability to misleading Italian symbols. We found that merely having Italian symbols on the package might lead almost half of the consumers in the sample to consider food as Made in Italy, regardless of the actual origin. This result confirms the severity of the problem. The econometric model provides suggestions for public actions to mitigate the issue.

## 1. Introduction

The Italian export of agri-food products has been increasingly threatened by the growth of various unfair practices using images of the “Made in Italy” branding and harming the performance of the Italian agri-food industry. The Made in Italy indication was born around the 1980s, when Italian products attracted high recognition all over the world for their quality and taste. As imitation by international competitors arose, it became necessary to identify the authentic Italian products from fake ones. Nowadays, the Made in Italy indication identifies a set of products and services for which Italy is renowned for their quality, innovation, design, and other elements [1] and which contribute to the international perception of the Italian style and the “Italian way of life”.

Made in Italy branding has proven to be an extraordinary export driver and a key source of competitive advantage, especially in the so-called ‘3-F industries’: fashion, furniture, and food. 

The Made in Italy indication makes food products more appealing and attractive to consumers because it is a quality clue [2], indicating not only the mere geographical origin, but also an added value that is grounded in tangible and intangible product attributes such as the quality of the ingredients, the Italian tradition, and local culture. All those elements support differentiation strategies based on the ‘uniqueness’ of Italian food. For this reason, the authenticity of the place of origin indication is a key issue for preserving the positive image of the Italian food and beverage industry and ultimately, its competitive advantage.

In order to explore this issue, we have designed a questionnaire to test the general knowledge of foreign (i.e., non-Italian) consumers about Made in Italy food and their association of Italian colors or images or wording with the actual Italian origin of food products. We investigated a sample of Spanish and English consumers, asking them about their perception of Italian food products and restaurants, with questions investigating the awareness of issues related to products that may seem Italian from the colors or the wording of distinctive signs or labels, but in reality they are not. In this way, we assessed the vulnerability of these consumers to unfair marketing strategies of non-Italian producers based on the misleading use of Italian symbols. 

## 2. Literature Review

According to Roth and Diamantopoulos [3], consumer perceptions of a product are related to the process of selecting, organizing, and interpreting intrinsic (i.e., organoleptic) or extrinsic (such as brand, price, origin) stimuli. In our case, we focused on the country of origin (COO) signal defined by a set of visual, verbal, or textual stimuli that consumers find on the product.

The concept of COO is strictly connected with the concept of the “country image”, which, in turn, has two effects on the consumer: the halo effect and the summary construct effect [4], These two effects depend on the level of knowledge that the consumer has about a certain country and the type of product under consideration. The halo effect applies when the level of knowledge of consumers on specific products/country is low, while the summary construct effect is usually applied when the level of knowledge of consumers is quite high. The country halo effect and summary construct effect refer to the relationship of the product and the COO in the minds of consumers.

According to Erickson et al. [5], the country halo effect emerges when the country image influences the perception of product attributes. When the consumer is not familiar with a product, the country image works as a halo from which they can deduce the product’s attributes without directly experiencing them (halo construct).

Instead, when the summary construct effect is in place, consumers associate product attributes to the country of origin [4]. The effect is achieved whenever the consumer is familiar with a country’s production, and associates the attributes experienced to the place of origin [6].

Firms can exploit the halo effect using a set of stimuli recalling a country (Italy, in this case) in order to lead consumers to project their perception of the country’s positive characteristics (e.g., Italian culture, taste, or way of life) on the product. If consumers are unfamiliar with the product, the transfer due to the halo effect can be an effective cue for quality [7]. Successful marketing strategies based on country image indirectly influence product preferences by eliciting product beliefs [8]. For example, in both emerging and developed countries, we have witnessed a growth in the desire to consume foods and drinks associated with the food and wine culture and identity of certain countries that the upper classes associate with style and elegance. The phenomenon of “desire of Europe” is particularly focused on the food and wine traditions of two countries: France and Italy [9]. The use of Italian symbols on food products originates from the profit potential of marketing strategies exploiting a very strong desire to consume foods and drinks associated with the food and wine culture and traditions of Italy [9]. The perception of Italian food has evolved in terms of cultural and social prestige over decades. Advertising, marketing, journals, magazines, and movies have played a crucial role in constructing a contemporary image about Italian food. Food traditions have been central in the success and to a certain level of idealization of Italian food, and have become strategic factors in generating popularity and commercial success [10]. Sometimes, however, it can be used to confuse the consumer, but not the consumer who has knowledge of the specific attributes of Italian products.

The meta-analysis study [11] on the link between country of origin and buyer behavior has shown that the COO concept is increasingly relevant in the marketing strategies of companies and territories [12]. These marketing actions are mainly aimed at highlighting and strengthening the link between the image of the place of origin (the country, the region, or the city) and the image of the products/services that are designed/manufactured in it. Moreover, the main input that emerged from this study is the lack of a clear and shared elaboration of the “country image” construct.

The use of “typically Italian” features from foreign companies can induce consumers to buy fake Made in Italy products because of the COO effect, even in those countries where Italian presence and culture have always been preserved. If the image of a country is perceived in a positive way by the target, a company can take advantage of their perception, using the image of the country as a way to add value to the product [13]. 

In the past, the country of origin and a good quality/price ratio were the key strategic assets for gaining and preserving international market shares. Nowadays, market shares are also increased by moving towards new product attributes, specifically environmental friendliness and food safety [14,15,16].

Through a case study on Italian food using typical Italian features conducted in Germany, and based on a survey, it was analyzed how the misleading use of Italian symbols affects the market, and whether consumers are aware of it, by investigating respondents’ perceptions about Made in Italy products and their purchasing behaviors [17]. The empirical analysis demonstrated that German consumers perceive Italian products as “high quality” and “very expensive” goods. These data show the up-market positioning of Made in Italy products, which can be considered as niche products. Moreover, it was found that consumers do not pay attention to the label on the product to understand the real origin, instead they basically choose an “Italian product” by looking at wordings, colors, and other symbols [18,19]. 

Bonaiuto, et al. [20] conducted three empirical studies in different cultural contexts (Italy, China, and the USA), finding differences in the attitude toward the products with Italian symbols and in purchasing behavior. The empirical analysis of the three studies found that Made in Italy products have comparative advantage in terms of the main dependent variables: reputation profile, general reputation, attitude, and willingness to pay (WTP). The analysis also provided psychological insights, suggesting that the more a product is perceived to be Italian, the higher financial competitive advantage it has.

The Italian appearance of a foreign product is a worldwide issue affecting an extensive variety of economic sectors, and the agri-food one in particular. Although its economic impact has been widely debated, methodical scientific knowledge concerning its social drivers is lacking [20]. If foreign consumers do not have information or knowledge about Italian food products, they may support their purchase using their perceived country image that is based on whatever knowledge they have about the country itself, even if not related to that specific product category. This consideration reinforces the motivation of this study because the positive reputation of Italy in the 3-F industries may extend to other sectors. Thus, the damage due to the unfair use of Italian symbols by foreign producers can be extensive.

## 3. Materials and Methods

The aim of this study was to investigate how foreign consumers assess whether a given food product is actually “Made in Italy” or not. We focused our attention on consumers from two areas: Spain and England, which have been selected due to the absence of previous studies on how Italian symbols affect purchasing decisions in these countries.

A survey was conducted using linear snowball sampling. The survey was administered online through the Google form platform in the months of October and November 2021. The link for the questionnaire was sent to the participants through social media channels: WhatsApp, Instagram, and Facebook, to individuals and to groups of Spanish and English “foodies” (i.e., people very interested in food, although not professionally involved in it). All respondents have consented to participation in the study.

We investigated how Italian symbols affect the choices of Spanish and English consumers. These factors were detected in the questionnaire.

Several symbols are placed on product packaging. The first evocative element is the name. The name can be related to the product name, such as “mozzarella cheese”, “Italian pizza”, etc., or it can be related to the brand name. In the latter case, common Italian first names (such as Luigi or Mario) or surnames are used as a reference to typical Italian culture and tradition. Other names commonly used are those of world-famous Italians. Other evocative elements are the writing on the packaging related to traditional Italian recipes or evocative phrases/adjectives that can lead customers to believe that those products are Italian.

Visual elements are important on the packaging because they represent the first impression that consumers have about products. Usually, they are related to colors or pictures on the label or packaging referring to Italy. The green, white, and red colors of the Italian flag and images representing famous Italian locations are among the most common visual elements. Lemons, basil, and the Italian boot shape are used as Italian symbols as well.

Additionally, we investigated consumers’ knowledge about instruments used to identify Italian restaurants abroad. The Authentico App not only provides the tools to foreign consumers to recognize the real Italian restaurants, but allows, by indicating the city where you are, to access a list of all the Italian restaurants and pizzerias in that city or specific area. Similarly, the certification Ita0039 is a certification of Italian restaurant authenticity that was introduced by the Italian Ministry of Agriculture.

The questionnaire used in this study consisted of 20 multiple-choice questions, 3 Likert scale questions, and a brief open-ended answer. To explore the different characteristics of the respondents, the questionnaire was divided conceptually into three sections.

The first section tested the respondents’ general knowledge of Made in Italy food and the Italian food sector. There were also questions about awareness of Italian food culture, information sources about Italian products (such as mass media or social media), and the role of advertising and word of mouth in identifying Italian food products.

In the second section, the perceptions of the Italian food and beverage industry were addressed. The objective of this section was to understand how foreign people perceive Italian food products and restaurants [21]. Questions investigated how consumers identify Italian food and their awareness of the possibility of buying fake Italian products. These items aim at understanding if and in which way the misleading Italian symbols have an impact on consumers’ perception and purchasing behavior. The questions concerned the role of certifications, the effect of visual symbols, the use of Italian names, and labels/packaging with Italian words on the food choices. The last section of the questionnaire was dedicated to the demographic data of the interviewees, reporting their age, gender, nationality, and their professional status.

## 4. Empirical Analysis

This research was conducted on primary data collected in Spain and England. A probit regression model was used for hypothesis testing. The literature review identified factors affecting knowledge of Italian food and perception of Italian symbols. The probit model allows to measure the impact of each factor on the probability of regarding a food product as Italian. The probit model is used when dichotomous output is influenced by independent variables.

The purpose of the model is to predict consumers’ vulnerability to the use of misleading Italian symbols, such as Italian flags, labels, words, or colors, by non-Italian producers. The probit model estimates the probability, P (Yi), that the response to the question “When you see Italian colors/flag on a food/beverage product, do you immediately trust this indication?” is positive.

The model explanatory variables are proxies for three main drivers affecting consumers’ vulnerability to misleading Italian symbols: (i) knowledge of Italian food and culture, (ii) self-assessment ability to identify authentic Italian food (including the use of apps or certifications), and (iii) main information sources. Demographic variables were used to capture any remaining systematic components in the model. Table 1 reports the variables that were used as proxies for the three drivers.

The final functional form is: P (Yi) = f (knowledge of fake products, ability to recognize fake product, visited Italy, advertising of Italian food products, information about Italian food products, knowledge of Italy, eating at Italian restaurants, personal knowledge, TV programs, through mass media, through social media, attention to label, Made in Italy as luxury food, Made in Italy as high quality, Certification 0039, frequency of eating in Italian restaurants, gender, age).

Specific representations of the probit model displaying the probability of choosing P may be expressed as:

(1) $$P{}_{i}=F(I_{i})=F\left(\right.\beta_{1}+\beta_{2}x_{i2}+\ldots \ldots \beta_{k}x_{ik}\left.\right)=F(x_{i}^{\prime }\beta )$$

(2) $$F\left(I_{i})\right.=P\left[z\leq I_{i}\right.]=\int \frac{1}{\sqrt{2\pi }}e^{-0.5z^{2\underset{}{}}}dz$$

where *z* is a standard normal random variable, the x’s are vectors of explanatory variables, and the β’s are parameters to be estimated. 

## 5. Results

A total of 104 questionnaires were returned. Table 2 reports the demographic characteristics of the sample. The distribution of the respondents in the convenience sample suggests that the data are balanced by gender and nationality. Elder consumers are underrepresented, probably because of the use of web-based survey tools. Although relatively small, the sample of our survey is large enough to grant our regression enough degrees of freedom (more than 70) to sustain a credible regression. We acknowledge that a small sample may result in inefficient (high variance) estimates of population means. Nevertheless, because regression parameters are unbiased estimators, we believe our model has explanatory power and can provide useful information to the reader.

In the sample, 51 out of 104 respondents (49%) answered that an Italian flag (or colors) on the packaging is a reliable identification of Italian products. The data confirm that a large share of consumers are possible targets for marketing strategies based on misleading Italian symbols.

Collected data were used to estimate the probit model (Table 3). A likelihood test of the hypothesis that all coefficients are 0 was performed and the hypothesis in question can be rejected. Therefore, it is possible to conclude that the proposed model has explanatory power. The model achieved 78.8% of correct predictions, and the area under the receiver operating characteristic (ROC) curve was 0.88. The statistics show that the model largely outperformed a prediction based on the sample average of the dependent variable alone. The Shapiro–Wilk test returned a z statistic of −0.593 and a *p*-value of 0.723, failing to reject the null hypothesis of normal distribution of residuals.

The results in Table 3 highlight the first important aspect: those who believe that there is a lot of advertising for Italian products in their country tend to trust food products that use colors or flags to evoke the product’s Italian origin. Based on this variable, it is sufficient to boast the Italian character of the product to convince the consumer about the product’s country of origin and take advantage of intensive umbrella promotions of Italian food. This result is of particular interest because it suggests that current publicly funded advertising campaigns promoting Italian food abroad may in fact benefit (and incentivize) fake products [22].

Trust in Italian symbols is conditional to the source of information that is used to identify authentic Italian food (regarding the role of the information source in food evaluation, also see [23]). Respondents who acquired information through Italian restaurants mistrusted Italian symbols the most. In fact, the coefficient of the variable showed a negative correlation with the probability of trusting symbols. Similarly, those consumers who stated that they have a personal culture of Italian food products did not rely on the colors or the images of the Italian flag shown on products. This variable was negatively correlated to the dependent variable and had a very high statistical significance.

The variable “high quality” was positively correlated with the trust in Italian symbols. This result suggests that a product marked with the colors of the Italian flag immediately gives foreign consumers the perception of a high-quality product. This was also confirmed by the “luxury products” variable, which was positively correlated with the dependent variable. 

Foreign consumers who knew of the Authentico App were likely to rely on Italian symbols to recognize an Italian product. This variable has a high significance. The result seems to contradict expectations because it suggests that consumers who are so concerned about authenticity to download (or at least be informed of) the app also trust Italian symbols. A possible explanation is that the possibility of using third-party certifications makes consumers more trusting.

Finally, the age variable played an interesting role. Millennials, Generation X, and Boomers are expected to be more vulnerable than Generation Z consumers (see in this regards [24]). All three age groups reported regression coefficients that were positive and statistically different from zero, showing that they trust Italian symbols more than the youngest consumers.

The variables that are associated with wariness of imitations are personal knowledge and knowledge through Italian restaurants. This means that the Italian products can be protected by in-depth personal knowledge of Italian food products. Existing literature [25] suggests that a strategy aimed at highlighting the authenticity of the origin of any product may not lead to any expansion in its market share. Products should be presented as part of a variety of traditional product packaging to promote food habits rather than just commodities.

## 6. Discussion

The matter of the protection of Italian food against fake products with Italian names has occupied center-stage in the recent public debate [26]. Our results justify this increasing concern about the unfair use of misleading Italian symbols.

The sample survey found that just having Italian symbols (such as the national flag or the national green, white, and red colors) on the package may lead a large share of consumers to believe that they are buying “Made in Italy” food. This result confirms the possible severity of the Italian appearance of food products and is in line with previous studies [27]. Extensive damage to the Italian food industry and to foreign consumers may result as a consequence [17]. 

The econometric model provides suggestions for public action to mitigate the issue. In particular, the effectiveness of mass media information campaigns is questionable. There is no statistical evidence that respondents who use TV or other mass media to obtain information about Italian products are less vulnerable to misleading use of Italian symbols. On the contrary, those who are exposed to Italian food advertising are more likely to trust Italian symbols. These results suggest that current information presented via mass media may not be effective in addressing the issue.

Looking at an analysis conducted on 750 adverts from the Dutch, British, and Spanish editions of Cosmopolitan, it emerged that the “made in” marker was rarely used. Instead, references to the COO in the company name and the use of COO language were most frequently employed. In total, 36% of the total number of advertisements contained at least one COO marker, and this underlines the role of the COO construct [28]. This result is in line with our study, and it suggests that public campaigns might consider focusing on consumer engagement instead of streaming advertising on mass media. In fact, results regarding information sources such as personal knowledge or Italian restaurants suggest that personal interactions with food and experts seem to be an effective approach to reduce the risk of buying products with misleading Italian symbols.

It must be noted that there is not statistical evidence to support that subjective assessment about one’s own ability to identify Italian appearance products from authentic Italian food is associated with a decrease in trust in Italian symbols. The regression coefficient was negative, but the t-statistics failed to reject the null hypothesis. Instead, the knowledge of specific tools such as the Authentico App was strongly associated with a reduced risk of buying fake Italian products. However, it must be noted that only 4% of the sample were aware of the Authentico App’s existence. This result suggests that consumers may not be able to self-assess their actual ability to detect Italian-sounding products, and some of them may be overconfident when dealing with the issue.

The recognition of products abroad is strongly associated with the knowledge of the products of a country. Knowledge about the culture of food could be a way to reduce the problem of counterfeiting and grant recognition to Italian products. This is in line with the study of Ricci [29]. In their research, consumers who had no personal experience with a product and that were unaware of its intrinsic attributes made their evaluation based on the products’ extrinsic attributes, and the COO was relevant to consumers that perceive it as a quality attribute. 

## 7. Conclusions and Recommendations for Businesses, Government, and Scholars

From a regulatory perspective, the Farm-to-Fork strategy of the European Commission is a milestone because the final document calls not only for the extension of a mandatory system of labeling the origin of all agri-food products at a European level, but also to guarantee the food traceability of food up to the final consumer, including retail and service sectors.

Of course, political action is just one way to protect Italian products. As emerged from our study, consumers that know Italian products and have a good knowledge of food culture have a higher probability of recognizing the real Italian food products, as well as those consumers that declare to recognize the Italian products thanks to their own personal knowledge.

Knowledge of the culture of food is a way to reduce consumers’ vulnerability to misleading Italian symbols and also to facilitate easier recognition of real Italian products. Consumers with limited food culture may be unaware of food products’ intrinsic attributes, make their evaluation based on products’ extrinsic attributes and easily fall for misleading symbols. In this regard, spreading the knowledge of Italian food culture might be a profitable investment for Italian food enterprises. 

Some indications for policymakers arose from the analysis carried out in this paper. It emerged that there is a need to provide foreign consumers with tools for food traceability, in order to guarantee them a conscious purchase and allow Italian producers to protect their authentic products. Digital innovation is a promising perspective in this regard, as suggested by our results about the use of the Authentico App. 

Finally, some limitations of the study should be considered. The study was based on a survey that was conducted on a self-selected sample of a relatively small number of consumers. The survey was sent to Spanish and English consumers, who then forwarded it to people they knew and to foodies; thus, people with a specific interest in the subject matter. The diversity within the sample was able to provide new insights into the issue, but the limitations also call for future studies and a deeper investigation with larger sample sizes in different countries.

The second limitation could be seen as the language used for the survey. The survey was sent to both Spanish and English people, but only in the English language. This represents a limitation for two main reasons: Spanish respondents with a low level of English may not properly understand the questions, and therefore may answer the questions following their intuition and not their real thoughts. The second reason is the fact that the use of the English language might have discouraged Spanish individuals whose English language levels were low from participating, resulting in a possible bias in the sample selection.

## Figures and Tables

**Table 1:** Definition of variables.

Variables	
Knowledge of fake Italian food products	1 = Yes, 0 = Otherwise
I cannot recognize a typical Italian product	1 = Yes, 0 = Otherwise
I can recognize a typical Italian product	1 = Yes, 0 = Otherwise
Italian foods advertised in your country (enough/a lot)	1 = Yes, 0 = Otherwise
Information about typical Italian products: MassMedia + TV	1 = Yes, 0 = Otherwise
Information about typical Italian products: Been to Italy	1 = Yes, 0 = Otherwise
Information about typical Italian products: Restaurant	1 = Yes, 0 = Otherwise
Information about typical Italian products: Personal knowledge	1 = Yes, 0 = Otherwise
When you buy a food product, do you pay attention to the label?	1 = Yes, 0 = Otherwise
Element related to Made in Italy: High quality	1 = Yes, 0 = Otherwise
Element related to Made in Italy: Luxury products	1 = Yes, 0 = Otherwise
Know of the Authentico App for authentic Italian products/restaurants	1 = Yes, 0 = Otherwise
Sex: Male	1 = Yes, 0 = Otherwise
Age: 26–35	1 = Yes, 0 = Otherwise
Age: 36–45	1 = Yes, 0 = Otherwise
Age: 46–55	1 = Yes, 0 = Otherwise
Age: More than 55	1 = Yes, 0 = Otherwise
Country: Spain	1 = Yes, 0 = Otherwise

**Table 2:** Demographic characteristics of the participants.

Age	Number	Percentage
18–25	39	37.50
26–35	39	37.50
36–45	11	10.58
46–55	11	10.58
>55	4	3.85
Total	104	100.00
**Gender**	**Number**	**Percentage**
female	63	60.58
male	41	39.42
total	104	100
**Nationality**	**Number**	**Percentage**
Spain	60	57.69
England	44	42.31
total	104	100

**Table 3:** Binary probit model results.

	Coeff.	Std. Err.	T-Stat	*p*-Value
Knowledge of fake Italian food products	−0.886	0.56	−1.583	0.113
I cannot recognize a typical Italian product	0.079	0.596	0.133	0.895
I can recognize a typical Italian product	−0.989	0.695	−1.423	0.155
**Italian foods advertised in your country (enough/a lot)**	**1.857**	**1.055**	**1.76**	**0.078**
Information about typical Italian products: MassMedia + TV	−1.348	0.962	−1.402	0.161
Information about typical Italian products: Been to Italy	−0.883	0.803	−1.099	0.272
**Information about typical Italian products: Restaurant**	**−1.815**	**0.963**	**−1.886**	**0.059**
**Information about typical Italian products: Personal knowledge**	**−4.971**	**1.873**	**−2.653**	**0.008**
When you buy a food product, do you pay attention to the label?	0.682	0.671	1.017	0.309
**Element related to Made in Italy: High quality**	**1.2**	**0.636**	**1.887**	**0.059**
Element related to Made in Italy: Luxury products	2.154	1.015	2.121	0.034
**Know of the Authentico App for authentic Italian products/restaurants**	**19.331**	**1.14**	**16.953**	**0.000**
Sex: Male	−0.303	0.559	−0.543	0.587
**Age: 26–35**	**1.119**	**0.588**	**1.902**	**0.057**
**Age: 36–45**	**2.025**	**0.805**	**2.514**	**0.012**
**Age: 46–55**	**2.567**	**0.984**	**2.61**	**0.009**
**Age: More than 55**	**1.725**	**1.038**	**1.662**	**0.097**
Country: Spain	−0.895	0.668	−1.34	0.18
Constant	−1.48	1.099	−1.347	0.178

Percent of correct predictions: 79%, the area under the ROC was 0.8818. Bold font indicates variables with a coefficient that is statistically different from zero at the 90% confidence level.

## Data Availability

The data presented in this study are available upon request from the corresponding author.
